# Adoption of Digital Technologies in Health Care During the COVID-19 Pandemic: Systematic Review of Early Scientific Literature

**DOI:** 10.2196/22280

**Published:** 2020-11-06

**Authors:** Davide Golinelli, Erik Boetto, Gherardo Carullo, Andrea Giovanni Nuzzolese, Maria Paola Landini, Maria Pia Fantini

**Affiliations:** 1 Department of Biomedical and Neuromotor Sciences University of Bologna Bologna Italy; 2 Department of Italian and Supranational Public Law University of Milan Milan Italy; 3 STLab ISTC-CNR Rome Italy; 4 IRCCS Istituto Ortopedico Rizzoli Bologna Italy

**Keywords:** COVID-19, SARS-CoV-2, pandemic, digital heath, review, literature, mitigate, impact, eHealth

## Abstract

**Background:**

The COVID-19 pandemic is favoring digital transitions in many industries and in society as a whole. Health care organizations have responded to the first phase of the pandemic by rapidly adopting digital solutions and advanced technology tools.

**Objective:**

The aim of this review is to describe the digital solutions that have been reported in the early scientific literature to mitigate the impact of COVID-19 on individuals and health systems.

**Methods:**

We conducted a systematic review of early COVID-19–related literature (from January 1 to April 30, 2020) by searching MEDLINE and medRxiv with appropriate terms to find relevant literature on the use of digital technologies in response to the pandemic. We extracted study characteristics such as the paper title, journal, and publication date, and we categorized the retrieved papers by the type of technology and patient needs addressed. We built a scoring rubric by cross-classifying the patient needs with the type of technology. We also extracted information and classified each technology reported by the selected articles according to health care system target, grade of innovation, and scalability to other geographical areas.

**Results:**

The search identified 269 articles, of which 124 full-text articles were assessed and included in the review after screening. Most of the selected articles addressed the use of digital technologies for diagnosis, surveillance, and prevention. We report that most of these digital solutions and innovative technologies have been proposed for the diagnosis of COVID-19. In particular, within the reviewed articles, we identified numerous suggestions on the use of artificial intelligence (AI)–powered tools for the diagnosis and screening of COVID-19. Digital technologies are also useful for prevention and surveillance measures, such as contact-tracing apps and monitoring of internet searches and social media usage. Fewer scientific contributions address the use of digital technologies for lifestyle empowerment or patient engagement.

**Conclusions:**

In the field of diagnosis, digital solutions that integrate with traditional methods, such as AI-based diagnostic algorithms based both on imaging and clinical data, appear to be promising. For surveillance, digital apps have already proven their effectiveness; however, problems related to privacy and usability remain. For other patient needs, several solutions have been proposed, such as telemedicine or telehealth tools. These tools have long been available, but this historical moment may actually be favoring their definitive large-scale adoption. It is worth taking advantage of the impetus provided by the crisis; it is also important to keep track of the digital solutions currently being proposed to implement best practices and models of care in future and to adopt at least some of the solutions proposed in the scientific literature, especially in national health systems, which have proved to be particularly resistant to the digital transition in recent years.

## Introduction

### Background

The COVID-19 pandemic, like all global crises in human history, is causing unprecedented health and economic disruptions in many countries. However, at the same time, this new situation is favoring the transition to digital solutions in many industries and in society as a whole. One example of this transition is education [1]; this entire sector, from primary schools to universities, has developed new strategies for teaching remotely, shifting from lectures in classrooms to live conferencing or web-based courses [2]. Similarly, health care organizations have responded to the COVID-19 pandemic through the rapid adoption of digital solutions and advanced technology tools. During a pandemic, digital technology can mitigate or even solve many challenges, thus improving health care delivery. Digital tools have been applied to address acute needs that have arisen as a direct or indirect consequence of the pandemic (eg, apps for patient tracing, remote triage emergency services). However, many of the solutions that have been developed and implemented during the emergency could be consolidated in the future, contributing to the definition and adoption of new digital models of care.

The list of new digital solutions is rapidly growing [3]. In addition to “video visits,” these options include email and mobile phone apps as well as use of wearable devices, chatbots, artificial intelligence (AI)–powered diagnostic tools, voice-interface systems, and mobile sensors such as smart watches, oxygen monitors, or thermometers. A new category of service is the oversight of persons in home quarantine and large-scale population surveillance. Telemedicine and remote consultation have already proven to be effective at a time when access to health services for patients who do not have COVID-19 or for patients with nonacute COVID-19 is prevented, impeded, or postponed. In fact, according to Keesara et al [4], instead of using a model structured on the historically necessary model of in-person interactions between patients and their clinicians through a face-to-face model of care, current health care services and patient assistance can be guaranteed remotely through digital technologies.

Before the COVID-19 pandemic, it was expected that digital transformation in health care would be as disruptive as the transformations seen in other industries. However, as discussed by Hermann et al [5] and affirmed by Perakslis [6], “despite new technologies being constantly introduced, this change had yet to materialize.” The spread of COVID-19 appears to have finally provided an ineludibly sound reason to fully embrace the digital transformation. Moreover, simulations show that many countries will probably face several waves of contagion, and new lockdowns will probably occur [7]. Therefore, it has become necessary to review the digital technologies that have been used during the emergency period and consider them for continued use over time or cyclically in the event of recurring outbreaks.

### Goal of This Study

According to Hermann et al [5], digital technologies can be categorized based on the patient needs they address in health care: diagnosis, prevention, treatment, adherence, lifestyle, and patient engagement. We argue that it is necessary to understand which digital technologies have been adopted to face the COVID-19 crisis and whether and how they can still be useful after the emergency phase. To achieve this, it is crucial to cover as many aspects as possible of digital technology use in health care in response to the COVID-19 pandemic.

The aim of this study is therefore to describe the digital solutions that have been reported in the early scientific literature to mitigate the impact of COVID-19 on individuals and health systems.

## Methods

### Literature Search

We conducted a systematic review of the early scientific literature, following the Preferred Reporting Items for Systematic Reviews (PRISMA) approach [8], to include quantitative and qualitative studies using diverse designs to describe which digital solutions have been reported to respond and mitigate the effects of the COVID-19 pandemic. This review focuses on health research, which includes biomedical, epidemiological, clinical, public health, and health systems research.

The initial search was implemented on May 11, 2020, and was limited to the timespan from January 1 to April 30, 2020. The search query consisted of terms considered adequate by the authors to review the literature on the use of digital technologies in response to COVID-19. Therefore, we searched the MEDLINE database using the following search terms and database-appropriate syntax:

(“COVID-19”[All Fields] OR “COVID-2019”[All Fields] OR “severe acute respiratory syndrome coronavirus 2”[Supplementary Concept] OR “severe acute respiratory syndrome coronavirus 2”[All Fields] OR “2019-nCoV”[All Fields] OR “SARS-CoV-2”[All Fields] OR “2019nCoV”[All Fields] OR ((“Wuhan”[All Fields] AND (“coronavirus”[MeSH Terms] OR “coronavirus”[All Fields])) AND (2019/12[PDAT] OR 2020[PDAT]))) AND (digital[Title/Abstract] OR technology[Title/Abstract])]

We also searched the COVID-19/SARS-CoV-2 section of medRxiv, a preprint server for health science papers that have yet to be peer-reviewed, for studies related to digital technologies, using the search string *COVID-19 digital technology* with the same timespan restriction applied to the MEDLINE search.

The search strategies and eligibility criteria used are provided in Multimedia Appendix 1.

### Study Selection and Data Collection Process

We included articles for review if they were studies with original data or results referring to digital tools or interventions for COVID-19 and if they addressed the needs of patients or health care systems in the evaluation.

An article was excluded if it was not a study with original results; it did not focus on digital solutions for COVID-19; the full text was not available; or it was not written in English.

A two-stage screening process was used to assess the relevance of the identified studies. For the first level of screening, only the title and abstract were reviewed to preclude waste of resources in procuring articles that did not meet the minimum inclusion criteria. The titles and abstracts of the initially identified studies were checked by two independent investigators (DG and EB). For the second level of screening, all citations deemed relevant after the title and abstract screening were procured for subsequent review of the full-text article.

A spreadsheet in Excel (Microsoft Corporation) was developed to extract study characteristics such as the paper title, journal, publication date, type of technology, and patient needs addressed. In particular, we categorized the retrieved papers according to patient needs (diagnosis, prevention, treatment, adherence, lifestyle, and patient engagement). For the categorization of patient needs, we adapted the definition by Hermann et al [5], which reports the concept of “customer needs addressed” by the health care industry, to identify the patient health needs addressed by digital technology during the early phase of the COVID-19 pandemic.

The definition of patient needs is reported in Table 1. We added “surveillance” as an additional patient need to those identified by Hermann et al [5] given the importance of early identification and confinement of patients with COVID-19 to preserve population health, and a category of “other” to include any needs that were not considered in the previous categories.

**Table 1:** Definitions of the patient needs addressed by digital technologies.

Patient need	Definition
Diagnosis	The process of determining which disease or condition explains a person's symptoms and signs [9]
Prevention	Preventing the occurrence of a disease (eg, by reducing risk factors) or by halting a disease and averting resulting complications after its onset [10]
Adherence	The degree to which a patient correctly follows medical advice [11]
Treatment	The use of an agent, procedure, or regimen, such as a drug, surgery, or exercise, in an attempt to cure or mitigate a disease [12]
Lifestyle	Adoption and sustaining behaviors that can improve health and quality of life [13]
Patient engagement	Actively involving people in their health and health care [14]
Surveillance	The continuous, systematic collection, analysis, and interpretation of health-related data needed for the planning, implementation, and evaluation of public health practice [15]
Other	All patient needs, addressed by digital technology, which are not included in the previous categories

We built a scoring rubric by cross-classifying the patient needs addressed by the technology (or technologies) reported in each article with the type of technology itself. We relied on the report “Assessing the impact of digital transformation of Health Services” by the Expert Panel on Effective Ways of Investigating in Health (EXPH) of the European Commission [16] to classify the types of digital technologies (ie, AI, big data, chatbots, electronic health records [EHRs], mobile apps, robotics, sensors, telehealth, and telemedicine), integrating it with terms found within the analyzed articles when necessary (ie, blockchain, Internet of Things [IoT], internet search engines, social media, and mobile tracing).

We also extracted information and classified each technology reported by the selected articles according to health care system targets, grade of innovation, and scalability to other geographical areas. To do this, we also relied on the classifications and definitions reported by the EXPH (Table 2) [16].

**Table 2:** Classification of digital technologies and health services.

Classification category		Definition
**Health care system targets**		
	Clients/patients	Members of the public who are potential or current users of health services, including caregivers.
	Health care providers	Members of the health care workforce who deliver health services.
	Health systems/resource managers	Systems and managers involved in the administration and oversight of public health systems. Interventions within this category reflect managerial functions related to supply chain management, health financing, and human resource management.
	Data services	Crosscutting functionality to support a wide range of activities related to data collection, management, use, and exchange.
**Grade of innovation**		
	Supporting	Digital services or technologies that can be used to support old or established technologies for all or some health care system targets. These technologies may support or facilitate the performance of existing technologies.
	Complementing	Digital services or technologies that can be used in addition to old or established technologies for all or some health care system targets. These technologies may strengthen or enhance the performance of existing technologies.
	Substituting	Digital services or technologies that may replace old or established technologies for all or some health care system targets.
	Innovating	New digital services or technologies that may offer new possibilities that previously were not available for all or some health care system targets. These disruptive technologies may represent a new entry into the market.
**Scalability to other geographical areas**		
	Not possible	Technologies strictly bonded to the context in which they were developed.
	Local	Technologies whose scalability is limited to a local context (ie, regional or national context) for normative, legislative, ethical, or technical reasons.
	Global	Technologies that do not present barriers to scalability that would prevent their possible global adoption.

Some of the analyzed articles described multiple technologies. For these articles, we reported all the health care system targets addressed by the proposed technologies. However, we found it impractical to assign different grades of innovation and scalability for each technology reported. Therefore, we chose to report only the highest grade of innovation or scalability assigned to the technologies within each article (eg, innovating>substituting>complementing>supporting).

Two of the authors (DG and EB) independently classified all identified articles in the predefined categories. Any disagreements were resolved through discussion and consensus between the two reviewers. If disagreement persisted, another reviewer (GC) was called as a tiebreaker.

Given the characteristics of this literature review, which aims to describe proposed digital solutions, and the nature and design of the included studies, assessments of the risk of bias and the study quality were not possible and therefore were not performed.

## Results

### Literature Search

**Figure 1:**
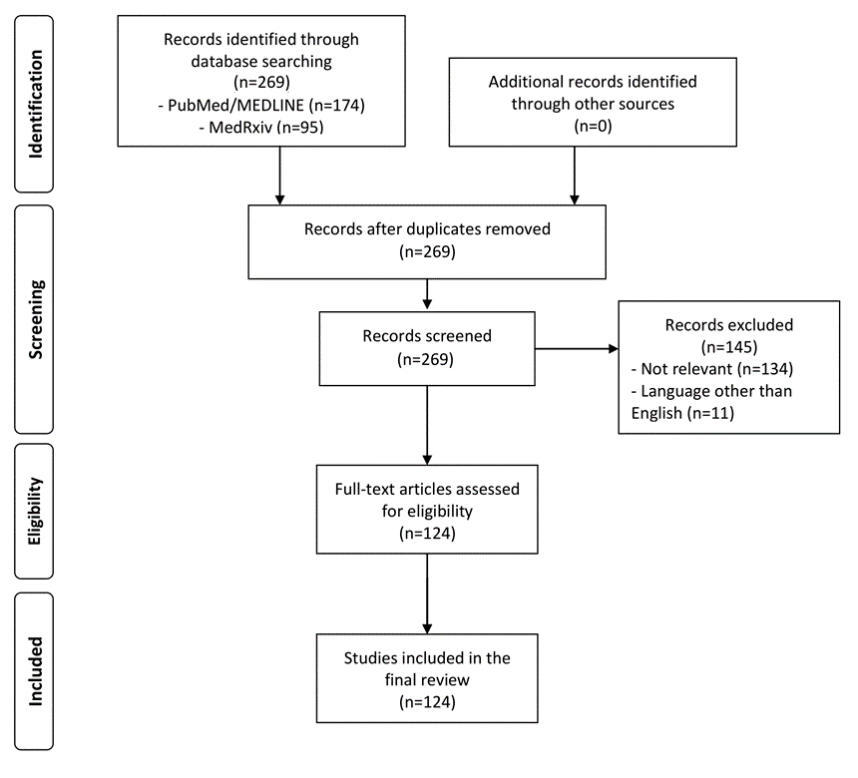
Preferred Reporting Items for Systematic Reviews (PRISMA) literature review flowchart.

The search identified 269 articles (174 from PubMed and 95 from medRxiv), of which 124 full-text articles were assessed and included in the review after screening (Figure 1).

### Study Selection and Data Collection Process

**Figure 2:**
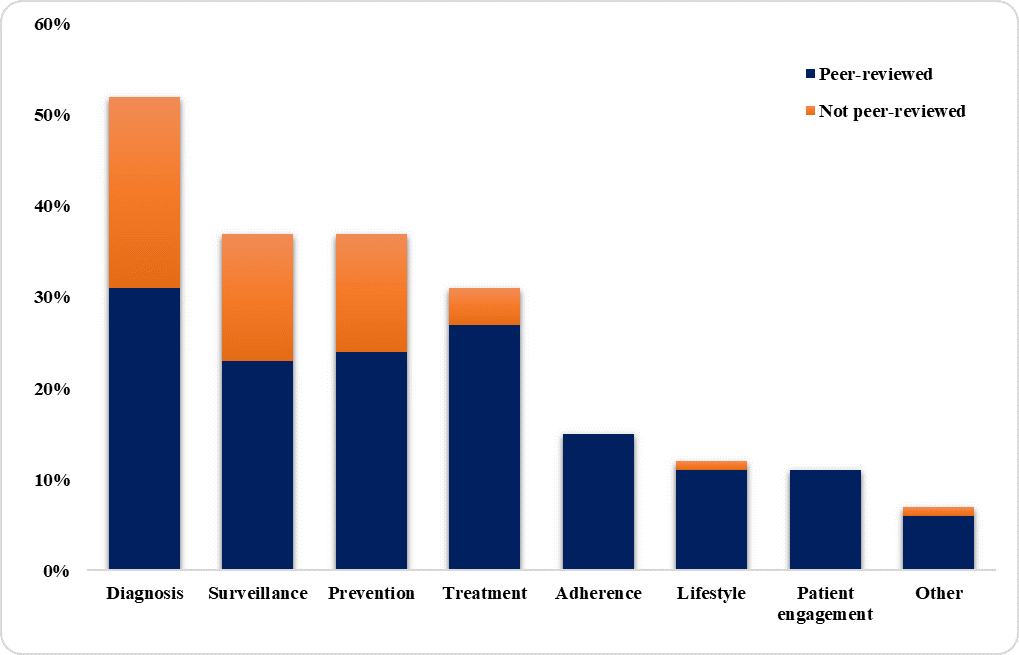
Frequency of appearance of each patient need within the 124 selected articles and the share of peer-reviewed articles for each need. The total percentage is higher than 100 because some articles include technologies used to address more than one patient need.

Out of the 124 selected articles, 65 (52.4%) addressed the use of digital technologies for diagnosis (Figure 2), 46 (37.1%) addressed surveillance, 46 (37.1%) addressed prevention, 38 (30.6%) addressed treatment, 15 (12.1%) addressed adherence, 12 (9.7%) addressed lifestyle, 11 (8.9%) addressed patient engagement, and 6 (4.8%) addressed other purposes. Considering the share of peer-reviewed articles, we found that for diagnosis, 39/65 articles (60%) were peer-reviewed; for surveillance, 29/46 (63%); for prevention, 30/46 (65%); for treatment, 33/38 (87%); for adherence, 15/15 (100%); for lifestyle, 11/12 (92%); for patient engagement, 11/11 (100%); and for other, 5 (83.3%).

In Table 3, we provide an extract of the characteristics of the included articles. An extended version of this table is provided in the supplementary material (Table S1, Multimedia Appendix 2).

**Table 3:** Articles included in the literature review with the main characteristics of each analyzed paper.

ID	Reference	Health care system targets	Grade of innovation	Scalability
1	Zhai et al [17]	Clients/patients, health care providers, health systems/resource managers, data services	Innovating	Global
2	Wang W et al [18]	Clients/patients, health care providers, health systems/resource managers, data services	Innovating	Local
3	Wang et al [19]	Clients/patients, health care providers, health systems/resource managers, data services	Innovating	Local
4	Yan et al [20]	Clients/patients, health care providers	Supporting	Global
5	Hou et al [21]	Clients/patients, health systems/resource managers, data services	Innovating	Local
6	Feng et al [22]	Health care providers	Complementing	Global
7	Jin et al [23]	Health care providers	Complementing	Global
8	Torous et al [24]	Clients/patients, health care providers, health systems/resource managers	Innovating	Global
9	Wang et al [25]	Health care providers	Complementing	Global
10	Zheng et al [26]	Health care providers	Complementing	Global
11	Galbiati et al [27]	Health care providers, health systems/resource managers	Supporting	Global
12	Bai et al [28]	Health care providers	Complementing	Global
13	Ienca et al [29]	Clients/patients, health systems/resource managers, data services	Innovating	Local
14	Ting et al [30]	Clients/patients, health care providers, health systems/resource managers, data services	Innovating	Global
15	Hua et al [31]	Clients/patients, health systems/resource managers, data services	Innovating	Local
16	Fu et al [32]	Health care providers	Complementing	Global
17	Zhou et al [33]	Health care providers	Complementing	Global
18	Lin et al [34]	Clients/patients, health care providers, health systems/resource managers, data services	Innovating	Local
19	Ferretti et al [35]	Clients/patients, health care providers, health systems/resource managers, data services	Innovating	Global
20	Calton et al [36]	Clients/patients, health care providers	Innovating	Global
21	Lin et al [37]	Health care providers	Complementing	Global
22	Mashamba-Thompson et al [38]	Clients/patients, health systems/resource managers, data services	Innovating	Local
23	Salako et al [39]	Clients/patients, health care providers	Innovating	Local
24	Hernández et al [40]	Clients/patients, health systems/resource managers	Innovating	Local
25	Ohannessian et al [41]	Clients/patients, health care providers, health systems/resource managers, data services	Innovating	Local
26	Shanlang et al [42]	Clients/patients, health systems/resource managers, data services	Innovating	Local
27	Turer et al [43]	Clients/patients, health care providers	Innovating	Global
28	Keesara et al [4]	Clients/patients, health care providers, health systems/resource managers, data services	Innovating	Local
29	Her [44]	Clients/patients, health systems/resource managers, data services	Innovating	Local
30	Klum et al [45]	Clients/patients, health care providers	Supporting	Global
31	Calvo et al [46]	Clients/patients, health systems/resource managers, data services	Innovating	Local
32	Dandekar et al [47]	Health systems/resource managers, data services	Innovating	Global
33	Drew et al [48]	Clients/patients, health systems/resource managers, data services	Innovating	Local
34	Segal et al [49]	Clients/patients, health systems/resource managers, data services	Innovating	Local
35	Hassanien et al [50]	Health care providers	Complementing	Global
36	Martin et al [51]	Clients/patients, health care providers, health systems/resource managers, data services	Innovating	Local
37	Yasaka et al [52]	Clients/patients, health care providers, health systems/resource managers, data services	Innovating	Local
38	Medford et al [53]	Clients/patients, health systems/resource managers, data services	Innovating	Local
39	Salg et al [54]	Health systems/resource managers, data services	Complementing	Local
40	Abhari et al [55]	Health systems/resource managers, data services	Innovating	Global
41	Jarynowski et al [56]	Clients/patients, health systems/resource managers, data services	Innovating	Local
42	Stommel et al [57]	Clients/patients, health care providers	Complementing	Global
43	Judson et al [58]	Clients/patients, health care providers, health systems/resource managers, data services	Innovating	Local
44	Ćosić et al [59]	Clients/patients, health care providers	Innovating	Local
45	Grange et al [60]	Clients/patients, health care providers	Innovating	Global
46	Castiglioni et al [61]	Clinets/patients, health care providers	Complementing	Global
47	Serper et al [62]	Clients/patients, health care providers	Innovating	Global
48	Robbins et al [1]	Clients/patients, health care providers, health systems/resource managers, data services	Innovating	Local
49	Crump [63]	Clients/patients, health care providers, health systems/resource managers, data services	Innovating	Global
50	Punn et al [64]	Health systems/resource managers, data services	Innovating	Global
51	Myers et al [65]	Clients/patients, health care providers, health systems/resource managers, data services	Innovating	Global
52	Noonan et al [66]	Clients/patients, health care providers, health systems/resource managers, data services	Innovating	Local
53	Loebet et al [67]	Clients/patients, health care providers, health systems/resource managers, data services	Innovating	Global
54	Price et al [68]	Health care providers	Innovating	Local
55	Vaishya et al [69]	Health care providers	Complementing	Global
56	Stubblefield et al [70]	Health care providers	Complementing	Global
57	Yuan et al [71]	Clients/patients, data services	Innovating	Local
58	Pollock et al [72]	Clients/patients, health care providers, health systems/resource managers, data services	Innovating	Local
59	Mahmood et al [73]	Clients/patients, health care providers, health systems/resource managers, data services	Innovating	Local
60	Gallotti et al [74]	Clients/patients, data services	Innovating	Local
61	Ren et al [75]	Clients/patients, health care providers, health systems/resource managers, data services	Innovating	Global
62	Goldschmidt [76]	Clients/patients, health care providers	Innovating	Global
63	Al-karawi et al [77]	Health care providers	Complementing	Global
64	Kumar et al [78]	Health care providers	Complementing	Global
65	Garg et al [79]	Clients/patients, health care providers, health systems/resource managers, data services	Innovating	Global
66	Kuziemski et al [80]	Health systems/resource managers, data services	Innovating	Local
67	Jakhar et al [81]	Clients/patients, health care providers	Innovating	Local
68	Marasca et al [82]	Clients/patients, health care providers	Innovating	Local
69	Bulchandani et al [83]	Clients/patients, health systems/resource managers, data services	Innovating	Local
70	Green et al [84]	Clients/patients, health care providers, health systems/resource managers, data services	Innovating	Local
71	Nagra et al [85]	Clients/patients, health care providers	Innovating	Global
72	O'Connor et al [86]	Clients/patients, health care providers, health systems/resource managers, data services	Innovating	Local
73	Wittbold et al [87]	Clients/patients, health care providers	Innovating	Local
74	Hightow et al [88]	Clients/patients, health care providers	Innovating	Local
75	Bonavita et al [89]	Clients/patients, health care providers	Innovating	Local
76	Wosik et al [90]	Clients/patients, HO	Innovating	Global
77	Yan et al [91]	Health care providers, health systems/resource managers, data services	Supporting	Global
78	Lin et al [92]	Clients/patients, health care providers, health systems/resource managers, data services	Supporting	Not possible
79	Kummitha [93]	Clients/patients, health care providers, health systems/resource managers, data services	Innovating	Global
80	Abbas et al [94]	Health care providers, data services	Complementing	Global
81	Ardabili et al [95]	Health systems/resource managers, data services	Supporting	Global
82	Zhang et al [96]	Clients/patients, health care providers,	Supporting	Global
83	Hart et al [97]	Clients/patients, health care providers	Innovating	Global
84	Parikh et al [98]	Clients/patients, health care providers	Innovating	Global
85	Rahman et al [99]	Clients/patients, health care providers, health systems/resource managers, data services	Innovating	Global
86	Alwashmi Hart [100]	Clients/patients, health care providers, health systems/resource managers, data services	Complementing	Global
87	Sedov et al [101]	Health care providers, health systems/resource managers, data services	Complementing	Global
88	Mahapatra et al [102]	Health care providers; health systems/resource managers	Innovating	Global
89	Azizy et al [103]	Clients/patients, health care providers, health systems/resource managers, data services	Innovating	Global
90	Madurai et al [104]	Clients/patients, health care providers, health systems/resource managers, data services	Innovating	Global
91	Park et al [105]	Clients/patients, health care providers, health systems/resource managers, data services	Innovating	Local
92	Negrini et al [106]	Clients/patients, health care providers	Complementing	Global
93	Tanaka et al [107]	Clients/patients, health care providers	Complementing	Global
94	Randhawa et al [108]	Data services	Innovating	Global
95	Javaid et al [109]	Clients/patients, health care providers, health systems/resource managers, data services	Supporting	Global
96	Kyhlstedt et al [110]	Clients/patients, health care providers, health systems/resource managers, data services	Supporting	Global
97	Barbosa et al [111]	Health systems/resource managers, data services	Innovating	Global
98	Blake et al [112]	Clients/patients, health systems/resource managers	Supporting	Global
99	Reeves et al [113]	Clients/patients, health care providers, health systems/resource managers, data services	Substituting	Global
100	Khan et al [114]	Clients/patients,	Substituting	Global
101	Whelan et al [115]	Clients/patients, health care providers, health systems/resource managers	Complementing	Global
102	Meinert et al [116]	Clients/patients, health care providers, data services	Innovating	Global
103	Ekong et al [117]	Clients/patients, health care providers, health systems/resource managers	Supporting	Local
104	Pérez Sust et al [118]	Clients/patients, health care providers, health systems/resource managers, data services	Complementing	Global
105	Kim et al [119]	Clients/patients, health care providers, health systems/resource managers, data services	Complementing	Global
106	Krukowski et al [120]	Clients/patients, health care providers, health systems/resource managers	Complementing	Global
107	Liu et al [121]	Health care providers, health systems/resource managers	Supporting	Global
108	Vaid et al [122]	Health care providers, health systems/resource managers	Complementing	Local
109	Lee [123]	Clients/patients, health care providers	Supporting	Global
110	Ramsetty et al [3]	Clients/patients, health care providers, health systems/resource managers	Complementing	Global
111	Tarek et al [124]	Health systems/resource managers	Complementing	Global
112	Awasthi et al [125]	Health care providers, health systems/resource managers	Supporting	Global
113	Khan et al [126]	Health care providers, health systems/resource managers	Supporting	Global
114	Husnayain et al [127]	Health care providers, health systems/resource managers	Supporting	Global
115	Weemaes et al [128]	Health care providers, health systems/resource managers, data services	Innovating	Local
116	Espinoza et al [129]	Clients/patients, health care providers, health systems/resource managers	Innovating	Global
117	Shweta et al [130]	Health care providers, health systems/resource managers, data services	Complementing	Global
118	Brat et al [131]	Health care providers, health systems/resource managers, data services	Supporting	Local
119	Hegde et al [132]	Clients/patients, health care providers	Innovating	Global
120	Tobias et al [133]	Clients/patients, health care providers, health systems/resource managers, data services	Supporting	Local
121	Compton et al [134]	Clients/patients, health care providers, health systems/resource managers	Complementing	Global
122	Smith et al [135]	Health care providers, health systems/resource managers	Complementing	Local
123	Kalteh et al [136]	Health care providers, health systems/resource managers, data services	Supporting	Global
124	Woo et al [137]	Clients/patients, health care providers	Complementing	Global

In Table 4, we cross-classify the use of technology and the type of technology. As an example, 24 articles describe the use of AI tools for the diagnosis of COVID-19, while 34 describe the use of telehealth or telemedicine for treatment purposes. All included articles and related analyses are reported in Multimedia Appendix 2 (Table S1).

**Table 4:** Cross-classification of the published studies by the type of technology and the patient needs addressed by the technology.

Technology	Diagnosis		Surveillance		Prevention		Treatment		Adherence		Lifestyle		Patient engagement		Other	
	n	Refs^a^	n	Refs	n	Refs	n	Refs	n	Refs	n	Refs	n	Refs	n	Refs
Artificial intelligence	24	[20,22,23,25,26, 28,30,32,33,38, 48,50,54,61,69, 70,77,78,94,100, 108,109,122,130]	12	[30,38,48,64,93, 95,100,104,109, 116,122,126]	11	[30,38,47,55,64, 95,100,109, 116,122,126]	2	[102,109]	1	[109]	1	[109]	1	[116]	4	[80, 104, 108, 125]
Big data analytics	6	[30,54,100,109, 118, 122]	11	[19,29,30,34,42, 93,100,109, 122,126, 136]	12	[19,30,34,42,55, 100,109,118, 122,124, 126,136]	2	[109,118]	2	[109,118]	3	[40, 109, 118]	0	N/A^b^	2	[111, 128]
Blockchain	2	[30,38]	2	[30,38]	2	[30,38]	0	N/A	0	N/A	0	N/A	0	N/A	0	N/A
Chatbot	3	[51,58,129]	0	N/A	1	[51]	0	N/A	0	N/A	0	N/A	0	N/A	0	N/A
Electronic health records	7	[1,60,91,113,118, 130,131]	5	[1,37,91,113,133]	4	[113,118,131,133]	5	[1,60,91, 113,118]	1	[118]	1	[118]	0	N/A	0	N/A
Internet of Things	3	[92,103,109]	5	[92,93,99, 104,109]	3	[92,103,109]	2	[103,109]	1	[109]	1	[109]	0	N/A	1	[104]
Internet search engines, social media	1	[1]	8	[1,18,21,31,42, 56,74,127]	3	[18,42,127]	1	[1]	0		1	[53]	0	N/A	1	[121]
Mobile app	8	[85,87,88,91,92, 96,100,110]	8	[18,91-93,96,100, 110,116]	9	[18,71,88,92,96, 100,110,115,116]	3	[85,88,91]	3	[88,112, 115]	2	[71, 112]	3	[112,115, 116]	0	N/A
Mobile tracing	6	[48,49,73,100, 110,118]	14	[31,35,44,46,48, 49,52,73,83,100, 105,110,117,119]	6	[44,73,83,100,110, 118]	2	[73,118]	1	[118]	1	[118]	0	N/A	0	N/A
Robotics, mechanical tools, drones, sensors, wearable devices	5	[45,101,109, 114,132]	3	[101,104,109]	3	[101,109,114]	2	[27,109]	3	[45,109, 114]	1	[109]	1	[114]	1	[104]
Telehealth, tele-medicine	29	[1,17,39,57,59, 60,62,63,65-67, 72,73,75,76,81, 84-91,100,103, 107,118,120]	7	[1,73,75,89-91, 100]	18	[24,39,41, 43,71,73, 75,88-90, 100,103,115, 118,120,134, 135,137]	34	[1,3,17, 24,36,39, 57,59,60, 62,63,65 -68,72,73, 75,76,79, 84-86,88, 91,97,98, 103,106,107, 118,123,134, 135]	11	[24,82,88, 97,98,115, 118,120,134, 135,137]	8	[24, 65, 66, 71, 76, 79, 118, 137]	8	[59,76, 82,97, 115,120, 123,134]	0	N/A

^a^Refs: references.

^b^N/A: not applicable.

### Characteristics of the Digital Health Technologies Favored by the COVID-19 Pandemic

Given the heterogeneity of the included technologies and solutions, we summarized the findings through a narrative synthesis. In fact, some patient needs share the same technology for health issues, which can be considered superimposable (eg, prevention and surveillance are often addressed by the same technology, such as mobile surveillance apps); this is also evident from Table 4, where many articles report technologies addressing more than one health need. Accordingly, in the following paragraphs, we describe the digital technologies and solutions starting from the patient needs they are designed to mitigate. We then discuss some of the retrieved articles that we deemed most interesting for each patient need.

#### Diagnosis

In the early scientific literature, digital solutions and innovative technologies were mainly proposed for the diagnosis of COVID-19. In particular, within the reviewed articles, we identified numerous suggestions of the use of AI-powered tools for the diagnosis and screening of SARS-CoV-2 or COVID-19, as reported in Table 4 [20,22,23,25,26,28,30,32,33, 38,48,50,54,61,69,70,77,78,94,100,108,109,122,130]. Most studies propose the adoption of AI tools based on the use of computed tomography (CT) data [23,28,33]. For example, Zhou et al [33] developed and validated an integrated deep learning framework on chest CT images for autodetection of novel coronavirus pneumonia (NCP), with particular focus on differentiating NCP from influenza pneumonia (IP), ensuring prompt implementation of isolation. Their AI model potentially provides an accurate early diagnostic tool for NCP. This type of diagnostic tool can be useful during the pandemic, especially when tests such as nucleic acid test kits are short in supply, which is a common problem during outbreaks. However, performing CT scans as a screening method presents significant limitations, considering the risks of both radiation exposure and operator or machine-type dependence [25].

In addition to these studies, many authors proposed AI-powered diagnostic tools for COVID-19 that are not based on CT scan data [22,51,58]. Feng et al [22] developed and validated a diagnosis aid model without CT images for early identification of suspected COVID-19 pneumonia on admission in adult fever patients and made the validated model available via a web-based triage calculator that must be supplied with clinical and serological data (eg, age, % monocytes, interleukin-6). Similarly, Martin et al [51] proposed a chatbot and a symptom-to-disease digital health assistant that can differentiate more than 20,000 diseases with an accuracy of more than 90%. The authors tested the accuracy of the digital health assistant to identify COVID-19 using a set of diverse clinical cases combined with case reports of COVID-19, and they reported that the digital health assistant can accurately distinguish COVID-19 in 96% of clinical cases.

A further innovative digital technology proposed to support the diagnosis of COVID-19 is blockchain (or distributed ledger) technology. In one study [38], the authors recommended low-cost blockchain and AI-coupled self-testing and tracking systems for COVID-19 and other emerging infectious diseases in low- and middle-income countries. They developed a low-cost blockchain app that requests a user’s personal identifier before opening pretesting instructions. Following testing, the user uploads their results to the app, and the blockchain and AI systems enable the transfer to alert the outbreak surveillance. These types of solutions can also be of interest in high-income countries.

Another interesting digital tool proposed for the diagnosis and triage of patients is chatbots. Chatbots are applications that provide information through conversation-like interactions with users; they can be used for a broad range of purposes in health care (eg, patient triage, clinical decision support for providers, directing patients and staff to appropriate resources, and mental health applications). Chatbots can help evolve triage and screening processes in a scalable manner [129] and, as institutions become increasingly familiar with these tools, can be repurposed in the future for other public health emergencies as well as for more standard care uses.

#### Prevention and Surveillance

Our literature review suggests that digital technologies can be useful for COVID-19 diagnosis as well as for implementing prevention and surveillance measures.

Judson et al [58] deployed the Coronavirus Symptom Checker, a digital patient-facing self-triage and self-scheduling tool, to address the COVID-19 pandemic; it provides patients with 24-hour access to personalized recommendations, and it improves ambulatory surge capacity through self-triage, self-scheduling, and avoidance of unnecessary in-person care. The majority of patients who used this checker did not make any further contact with the health care system during subsequent days.

Another topic of paramount importance in the context of health care digitalization is epidemiological surveillance. Our review highlights that prevention and surveillance are often considered together in the scientific literature, given that “prevention of COVID-19” can be intended as “prevention of further spread,” which is mainly achieved through surveillance. For the COVID-19 pandemic, surveillance definitely overlaps with prevention, as the risk of infection can be reduced by applying a successful surveillance plan and controlling the interactions between infected persons and the healthy population.

A study by Ferretti et al [35] analyzed the key parameters of the COVID-19 epidemic spread to estimate the contributions of different transmission routes and determine the requirements for successful case isolation and contact tracing. The viral spread is too rapid to be contained by manual contact tracing. The solution is the implementation of a contact-tracing app that creates a temporary record of proximity events between individuals and immediately alerts recent close contacts of diagnosed cases and prompts them to self-isolate. An important limitation of this type of tracing technology is that to achieve its goal, it must be used by a significant portion of the population.

An example of successful use of a mobile app for contact tracing is the app that the Chinese government implemented in Wuhan [31]. Quick response (QR) code–based screening was implemented in Hubei Province to monitor people’s movement, especially on public transportation. Using big data and mobile phones, three color codes were attributed to each citizen: green (safe), yellow (need to be cautious), and red (cannot enter). A similar tool was implemented in Taiwan [19]. In fact, through Taiwan citizens’ household registration system and foreigners’ entry cards, it was possible to track individuals at high risk of COVID-19 infection because of their recent history of travel to affected areas. If an individual was identified as high risk when in quarantine, they were monitored electronically through their mobile phone. Then, the Entry Quarantine System was launched: through the completion of a health declaration form (requiring the scan of a QR code that leads to a web-based form, either prior to departure from or upon arrival at a Taiwan airport), travelers could receive rapid immigration clearance.

Our literature review suggests that another meaningful way to control the spread of an epidemic is through monitoring and surveillance of internet searches and social media usage. Wang et al [18] used WeChat, a Chinese social media platform, to plot daily data on the frequencies of keywords related to SARS-CoV-2. The authors found that the frequencies of several keywords related to COVID-19 showed abnormal behavior during a period ahead of the outbreak in China, and they stated that social media can offer a new approach to early detection of disease outbreaks. Similarly, the Italian words for “cough” and “fever” were searched in Google Trends to find useful insights to predict the COVID-19 outbreak in Italy, showing a significant association with hospital admissions or deaths in the two following weeks [138]. These two papers show that tracking public health information from web-based search engines may have a role in the prediction of future COVID-19 outbreaks, complementarily to traditional public health surveillance systems.

Furthermore, a technology that can aid the automatic, decentralized, and remote collection of data for surveillance purposes is the IoT. In [99], an IoT-based smart disease surveillance system showed potential to control the pandemic. In fact, with most people using smartphones and wearable technologies and having internet access, this technology can help limit the spread of the pandemic through the collection and analysis of default gathered data.

Although its potential is irrefutable, the technology behind surveillance and contact tracing apps raises many concerns; as discussed by Calvo et al [46], the most obvious concern is “surveillance creep,” which occurs when a surveillance tool developed for a precise goal (in the case of China and Taiwan, an app to monitor people’s movement) remains in use after the crisis is solved. Privacy must be a primary concern for policy makers and a key challenge for designers and engineers who design digital tools for epidemic control. As already outlined in previous work by Carullo [139], in the European Union, applications to combat COVID-19 should avoid processing personal data whenever possible. The General Data Protection Regulation (GDPR) [140] dictates the principle of privacy by default, that is, “by default, only personal data which are necessary for each specific purpose of the processing are processed.” In this regard, it should be reminded that according to the GDPR, data are “personal” only when and insofar they allow the identification of a natural person. Therefore, the processing of data, including clinical data, that cannot in any way identify a natural person does not involve personal data. Therefore, any privacy concerns are completely ruled out. To be compliant with this principle, a preferable approach is therefore to trace the spread of the virus, and therefore alert users, without collecting any personal data. A promising example in this direction was provided by Yasaka et al [52], who described an open-source proof-of-concept app for contact tracing that does not require registration or the divulgation of any private data, such as location. Instead, this tool uses an ingenious “checkpoint” system that allows users to create a peer-to-peer network of interactions and to determine if they have been exposed to any risk of infection; diagnosis of infection can be entered into the app, and the data are transferred to a central server but remain anonymous.

Although the aforementioned articles addressed surveillance and prevention in outpatients and the general population, an interesting point of view on inpatient surveillance comes from the study by Lin et al [37]. This paper describes a prospective active surveillance system with information technology (IT) services (ie, using a surveillance algorithm based on data from EHRs) to identify hospital inpatients whose pneumonia did not show marked improvement with antibiotic treatment and to alert the primary care medical teams on a daily basis. Similarly, EHR-based rapid screening processes, laboratory testing, clinical decision support, reporting tools, and patient-facing technology related to COVID-19 can be implemented using the EHR to build multiple COVID-19–specific tools to support outbreak management, including scripted triaging, electronic check-in, standard ordering and documentation, secure messaging, real-time data analytics, and telemedicine capabilities [113].

In the field of prevention, other important digital technologies proposed in the literature include telemedicine and telehealth. Telemedicine is not always applicable in emergencies, and many patients with COVID-19 may need to go to the hospital to receive higher level care. For this purpose, Turer et al [43] proposed using electronic personal protective equipment (ePPE) to protect staff (ie, prevent infection of health care workers) and conserve traditional PPE while providing rapid access to emergency care for low-risk patients during the COVID-19 pandemic. A very similar solution has been proposed by Wittbold et al [87]. They explored digital care delivery methods to reduce unnecessary exposure and conserve PPE through the deployment of iPads equipped with an app to evaluate and manage patients in a quaternary care academic and level one trauma center. Therefore, ePPE has been proved to be potentially applicable to settings such as emergency medical services, medical wards, and intensive care units.

#### Treatment and Adherence

Telemedicine and telehealth technologies are also used to increase patient adherence and for treatment purposes. Torous et al [24] describe the potential of digital health to increase access to and quality of mental health care by exploring the success of telehealth during the present crisis and how technologies such as apps can soon play larger roles. Telehealth is seen as a useful solution to deliver mental health care in general [141] and during social distancing and quarantine periods. In addition, digital therapy programs can be offered through courses of evidence-based therapies or using augmented reality and virtual reality systems. As another example, Calton et al [36] deliver some useful advice on the implementation of telemedicine to deliver specialty palliative care in the homes of seriously ill patients and their families. The authors state that the digital divide must be taken into account. Patients require access to an internet connection and to a digital device suited for videoconferencing. For older people or those less familiar with technology, it may be necessary to identify a caregiver as a “technological liaison” for the patient. To create a successful telemedicine-based treatment environment, many critical factors must be considered, including workforce training, high-quality evidence, digital equity, and patient adherence.

#### Lifestyle and Patient Engagement

In the early literature responding to the COVID-19 pandemic, fewer scientific contributions addressed the use of digital technologies for lifestyle empowerment or patient engagement. This is probably due to the current phase of the pandemic, which has conditioned scientific research to focus primarily on aspects related to more acute patient needs. However, some articles can be found. For instance, Krukowski et al [120] addressed the issue of remote obesity management through telehealth methodologies such as electronic scales to remotely measure patients’ weight and to maintain their engagement toward healthy lifestyles.

## Discussion

### Principal Results

Although SARS-CoV-2 is causing a pandemic worldwide, it is also favoring the rapid adoption of digital solutions and advanced technology tools in health care. On the one hand, physicians and health systems may need to track large populations of patients on a daily basis for surveillance purposes [4]. On the other hand, they may need rapid diagnostic tests for COVID-19 screening, to reduce the workload, and to enable patients to receive early diagnoses and timely treatments. This can also be achieved with the help of digital technologies, which were already available in different industries before the current crisis. These tools have now been quickly implemented in health care due to the pandemic [104].

In this systematic review of the early scientific literature in response to COVID-19, we describe numerous digital solutions and technologies addressing several patient and health care needs. The constantly updated scientific literature is a source of important ideas and suggestions for finding innovative solutions that guarantee patient care during and possibly after the COVID-19 crisis.

In the field of diagnosis, digital solutions that integrate with the traditional methods of clinical, molecular or serological diagnosis, such as AI-based diagnostic algorithms based both on imaging and clinical data, seem promising.

For surveillance, digital apps have already proven their effectiveness [142-144]; however, problems related to privacy and usability remain [145]. For other patient needs, several solutions have been proposed, such as telemedicine or telehealth tools. These tools have long been available; however, this historical moment could actually favor their definitive large-scale adoption.

The fact that the digital technologies proposed in the analyzed scientific literature mainly address the fields of diagnosis, prevention, and surveillance probably reflects the emergency phase of the COVID-19 pandemic. As time passes, well-known digital tools could be proposed for different purposes and patient needs, such as adherence, lifestyle, and patient engagement, which are considered to be important determinants of patient health [146] despite the lower attention paid to them in the early scientific literature.

In addition to the patient needs addressed by digital technologies, our review sheds light on the most used digital technology tools. Given the early phase of the pandemic and its reflection on the articles included in this review, the technologies that have shown to be more easily and quickly implementable can be also considered as the most scalable. In fact, the speed with which these technologies have been deployed demonstrates their ease of adoption and manageability in many different contexts, despite their deployment during the course of a pandemic. Many of these solutions have demonstrated a technical, economic, regulatory and usability weight that is sufficiently low to allow their rapid and effective use, at least during the emergency phase. Among these solutions, we report AI tools for diagnosis, big data analytics and mobile tracing for surveillance and prevention, and telemedicine and telehealth, which have proved to be transversal tools for diagnosis, prevention, and treatment.

We advocate that many of the digital technologies that have been quickly implemented in this emergency phase can also be adopted in the following phases of the pandemic, as also stated by Fagherazzi et al [147]. However, this implementation is easier said than done. According to the article by Keesara et al [4], “Covid-19 and Health Care’s Digital Revolution,” in the context of the digital leap caused by the COVID-19 pandemic in the United States (and worldwide) [4], while private corporations and education institutions have made rapid transitions to remote work and videoconferencing, the health care system is still lagging behind in adopting digital solutions. This is mainly due to the fact that clinical workflows and economic incentives have been developed for a face-to-face model of care that, during this pandemic, contributes to the spread of the virus to uninfected patients who are seeking medical care. In addition to the history of health care policies, there are limiting factors to the implementation of tools such as telemedicine, including a legal framework that is not yet fully designed to regulate the use of innovative IT systems in health care, as well as an inadequate information and communications technology infrastructure and an obsolete reimbursement and payment structure.

Many countries are facing these regulatory issues: the challenges for digital health have become a global issue in the public health response to COVID-19 and future outbreaks. Digital tools such as telemedicine should indeed be integrated into international and national guidelines for public health preparedness, alongside the definition of national regulations and funding frameworks in the context of public health emergencies. To switch to new digital models of care, increasing the digital expertise of health care professionals and educating the population are fundamental issues. Moreover, by implementing a data-sharing mechanism, digitally collected and stored data will be a precious tool for epidemiological surveillance that, as discussed earlier, is fundamental in controlling the epidemic spread. Lastly, to describe and assess the impact of digital tools during outbreaks, scientific evaluation frameworks should be defined [41].

### Limitations

This literature review presents some limitations. First, the research was conducted in a period of epidemiological emergency. Thus, the number of daily publications is high, and it is difficult to keep up to date. As a result, we have been forced to select articles in a reduced time span, potentially missing some studies and including studies that have yet to be peer-reviewed. Secondly, due to the design of the review, the search could not be fully comprehensive, as it was conducted exclusively on the MEDLINE database and medRxiv to preserve both time and resources; however, PubMed/MEDLINE is reported to be the primary database used by health science faculties [148], and medRxiv is one of the main repositories of COVID-19 research [149]. Finally, the articles and concepts included in this preliminary review certainly need to be integrated at the end of this international emergency phase.

### Conclusions

The COVID-19 pandemic is favoring the implementation of digital solutions with unprecedented speed and impact. It is therefore recommended to keep track of the ideas and solutions being proposed today to implement best practices and models of care tomorrow and to be prepared for future national and international emergencies. It is worth taking advantage of the impetus provided by the crisis we are currently experiencing to implement at least some of the solutions proposed in the scientific literature, especially in national health systems, which in recent years have proved to be particularly resistant to the digital transition.

## Appendix

Multimedia Appendix 1 Eligibility criteria and search strategy.

Multimedia Appendix 2 Table S1. Articles included in the literature review.

## Abbreviations

AI: artificial intelligence
CT: computed tomography
EHR: electronic health record
ePPE: electronic personal protective equipment
EXPH: Expert Panel on Effective Ways of Investigating in Health
GDPR: general data protection regulation
IoT: Internet of Things
IP: influenza pneumonia
IT: information technology
NCP: novel coronavirus pneumonia
PRISMA: Preferred Reporting Items for Systematic Reviews
QR code: quick response code
