# Supplementary material for: Adoption of Digital Technologies in Health Care During the COVID-19 Pandemic: Systematic Review of Early Scientific Literature
Source: J Med Internet Res. 2020 Nov 6;22(11):e22280. doi: 10.2196/22280 (PMC7652596; doi:10.2196/22280)
Supplement: Multimedia Appendix 1 [file jmir_v22i11e22280_app1.doc]

**Supplementary materials**

**Eligibility Criteria**

The inclusion criteria were as follows:

Population: The unit of analysis of this review is studies (i.e., study reports) published in health-related journals, including studies with original data or results.

Interventions/exposures: We included studies that describe digital solutions reported in the early scientific literature to mitigate the impact of COVID-19 on individuals and health systems.

Comparator: N/A

Outcome measures: To be included, studies had to report digital technologies or innovative solutions proposed or implemented to mitigate the impact of COVID-19 on individuals and health systems.

Study design: Experimental studies (eg, RCTs and nonrandomized studies or cohort studies), observational studies, case series, and case studies.

Publication type: We included original research papers, including scientific meeting abstracts or research letters, if they contained sufficient information to fill the extraction forms.

**Search strategy**

(search date: May 11, 2020)

| **Search strategy** | **Output** |
| --- | --- |
| ("COVID-19"[All Fields] OR "COVID-2019"[All Fields] OR "severe acute respiratory syndrome coronavirus 2"[Supplementary Concept] OR "severe acute respiratory syndrome coronavirus 2"[All Fields] OR "2019-nCoV"[All Fields] OR "SARS-CoV-2"[All Fields] OR "2019nCoV"[All Fields] OR (("Wuhan"[All Fields] AND ("coronavirus"[MeSH Terms] OR "coronavirus"[All Fields])) AND (2019/12[PDAT] OR 2020[PDAT]))) AND (digital[Title/Abstract] OR technology[Title/Abstract])] | MEDLINE (n= 174) |
| COVID-19 digital technology | MedrXiv (95) |
| **TOTAL** | **269** |
